# Insecure yet Resourceful: Psychological Capital Mitigates the Negative Effects of Employees’ Career Insecurity on Their Career Satisfaction

**DOI:** 10.3390/bs12120473

**Published:** 2022-11-24

**Authors:** Jetmir Zyberaj, Cafer Bakaç

**Affiliations:** 1 Work and Organizational Psychology Group, Department of Psychology, University of Bamberg, 96047 Bamberg, Germany; 2 TUM School of Management, Technical University of Munich, 80333 Munich, Germany

**Keywords:** career insecurity, psychological capital, career satisfaction, COVID-19

## Abstract

The COVID-19 pandemic has increased employee career concerns (i.e., insecurity), and many people face difficulties with their current jobs. In addition, employees have struggled with their health due to COVID-19. Based on the psychological capital (PsyCap) and the conservation of resource theories, we suggest that personal resources, such as resilience, can mitigate the adverse effects of employee career insecurity on their career-related outcomes, such as career satisfaction, as well as on their health. In a German-speaking sample (*N* = 185) and a two-wave design, we investigated the role of employees’ career insecurity on their career satisfaction. We employed PsyCap as a moderator in these relationships. Results showed a negative relationship between career insecurity and career satisfaction. In addition, moderation analyses revealed that PsyCap significantly moderates the effects of career insecurity on employee career satisfaction. Specifically, for high PsyCap the effect of career insecurity on employee career satisfaction does not hold significant, while it does for low PsyCap, showing that PsyCap can mitigate the negative effects of career insecurity on employee career satisfaction. With a robust personal construct in career research, our study contributes to this field by investigating the role of PsyCap for employee careers, especially in a crisis context (i.e., COVID-19). We discuss implications for employees and organizations.

## 1. Introduction

Job insecurity is an individual’s concern related to the “continued existence of the job in the future” [1] (p. 243). It can be among the worst job stressors [1,2], yielding negative psychological [3] and career [4] consequences. In addition to the various changes in the labor market, such as increased flexibility and technological advancements [5], the adverse effects of job insecurity have been reinforced by the current coronavirus disease 2019 (COVID-19). According to the Organisation for Economic Co-operation and Development (OECD), COVID-19 has caused unprecedented job losses, with a volume 10 times bigger than the global financial crisis of 2007–2008 [6]. Such events can have significant implications on employees’ career insecurity (i.e., a psychological dimension of job insecurity), which relates to one’s perception of a threat to their career mobility and progress [7].

With the implications of COVID-19 for workplaces, it is essential to add to the literature that can further explain the factors driving employees towards negative (or positive) evaluations of their careers [8,9]. Thus, with the increased career insecurities amidst COVID-19, we investigated employee career insecurity about their subjective career success, namely the subjective evaluations of one’s career progress [10] during the COVID-19 pandemic. Furthermore, to understand the ramifications of this career hurdle, we also looked into its effects on employees’ career worries caused by the COVID-19 pandemic, namely worries caused by one’s career hurdles [11]. Finally, to shed light on the role of stable individual differences for career success, we employed a personality concept as a possible moderator between career insecurity and subjective career success, namely the Psychological Capital or PsyCap [12]. Our major goal was to look into the possible role that PsyCap might play in employees’ career insecurity during a crisis. This is especially important because the PsyCap role informs us theoretically (e.g., about the role of personal characteristics for career development) and practically (e.g., informing relevant stakeholders about the implications such characteristics can yield for their career management systems).

PsyCap is a positive psychological resource comprised of hope, optimism, self-efficacy, and resilience [12,13]. Drawing on PsyCap [14] theory and the Conservation of Resource (COR, [15]) theory, we suggest that PsyCap mechanisms increase one’s subjective career success by mitigating the negative effects of career insecurity. For instance, Mithani (2020) noted that resilience helps employees to adapt during crises. Similarly, hope and optimism can help employees maintain their focus on the future through the temporal focus, which has been shown to correlate positively with coping with current and future tasks [16]. Recent research showed that PsyCap positively relates to employees’ subjective career success [17,18]. However, we know of no study that looked into the role of PsyCap in employees’ career insecurity, especially during crises.

With this study, we contribute to the existing research on job insecurity and subjective career success in two ways. First, we shed light on the relationship between employees’ career insecurity and career satisfaction, as well as career worries, thus filling this research gap. Second, accounting for the role of stable traits and personal resources for career success [19], we further enhance our understanding of such factors by employing PsyCap as a moderator in these relationships. This way, we provide insights into the role of the various personality characteristics in employees’ career insecurities and their subsequent subjective career success and worries in a crisis context.

## 2. Theoretical Foundations and Hypotheses Development

### 2.1. Career Insecurity

Career insecurity is a critical psychological dimension of job insecurity [7]. This construct is analyzed primarily in the context of boundaryless careers, characterized by mobility between occupations, industries, and employment forms [20]. According to Colakoglu [20], employees can experience career insecurity “when there is a perceived threat both to the continuity of one’s employability and to the quality of subsequent employment” (p. 48). The perceived threat causes employees to feel powerless, which can increase insecurity towards their employability, namely the subjective perception of their possibilities of obtaining and maintaining employment [21].

COR theory [15,22] posits that individuals are motivated to gain and preserve resources and avoid their loss, which can yield stress. Research has linked career insecurity with various negative work-related outcomes, such as work stress and turnover intentions [23,24]. According to COR theory, such negative events can increase, or decrease, people’s penchants “to retain, protect, and build resources” [15] (p. 516). However, as Hobfoll [22] reported, stress (i.e., due to perceived threat caused by career insecurity) related to a career is caused by the actual loss of resources and the perceived threat that resources might be lost. Such perceptions are reported to be mitigated by many personal and organizational factors [10,25,26]. For instance, the significance of employees’ self-beliefs (e.g., self-efficacy) is noted as crucial for career management and success [27,28]. Such factors are associated with better and improved subjective career successes [19,29].

### 2.2. Career Insecurity and Career Satisfaction

Career satisfaction concerns “the extent to which individuals believe their career progress is consistent with their own goals, values and preferences” [30] (p. 622). Based on COR theory [22], we assume that the extent employees feel subjectively satisfied with their careers depends on their resource acquisition and conservation, which are important for their careers, such as job or career satisfaction. However, in volatile economies and stressful environments, employees must strive to gain and conserve resources to manage their careers usefully [22,31,32]. Thus, when there is a threat to such resources, employees might experience resource loss and elevated levels of stress and insecurity [7,33,34], negatively affecting their career satisfaction [35,36]. Ng and Feldman [19] reported that resource loss can lead employees to experience less successful careers, which might yield lower career satisfaction. Hence, we hypothesize the following:

**Hypothesis** **1.** *Career insecurity is negatively related to career satisfaction.*

### 2.3. Career Insecurity and Career-Related COVID-19 Worries

Similar to career satisfaction, we expect that career insecurity also negatively affects employees’ career-related worries caused by COVID-19 [37,38]. Schwartz et al. (2000) defined worries as any cognitive representation of perceived threats. Because career insecurity implies reduced assurance and increased anxiety about one’s career success [20], we assume this increases their worries about their career during the COVID-19 pandemic [11,38]. This is also in line with the COR theory [22], which reports that loss of resources (i.e., following career insecurity) increases perceived threats (i.e., employee worries about their careers). Current research reports on COVID-19 as a career shock where factors such as “job insecurity, loss of income, the emotional impact of social distancing, and increased general anxiety” can increase the negative valence of this career shock and various perceived threats [39] (p. 119). With an increased perceived threat due to career insecurity during the COVID-19 pandemic, we assume employees will also report career worries. Hence, we conjecture the following:

**Hypothesis** **2.** *Career insecurity is positively correlated with career-related COVID-19 worries.*

### 2.4. Psychological Capital as a Moderator between Career Insecurity and Career Satisfaction

To draw on the ramifications of the PsyCap for career insecurity, we use the PsyCap theory as a theoretical framework [13]. Luthans et al. [40] defined PsyCap as “an individual’s positive psychological state of development” (p. 3), comprised of self-efficacy, optimism, hope, and resiliency. To explain the positive role of PsyCap, PsyCap theory uses three mechanisms: (1) malleability, (2) agency, and (3) sociability. According to Youssef-Morgan and Luthans [13], malleability facilitates individuals’ experiences through change and adaptation. Similarly, through agency, individuals strive to proactively influence their environment and alter the course of their actions per their plans and goals. Finally, with the help of sociability, individuals use social support as additional resources to pursue their goals.

Hobfoll et al. [15] posited that PsyCap resources can boost each other by acting as a “caravan” (i.e., acting jointly). Similarly, Youssef-Morgan and Luthans [14] noted that PsyCap mechanisms can facilitate employees’ coping with uncertain environments by working as a single body (naming them “HERO”). Different from previous moderators, such as attitudes [26] or big five [41] for employee careers, the role of PsyCap is particularly important due to the assumptions that the four mechanisms should act as a single body within individuals [12,14]. Factors such as the big five are often studied separately for their roles [41]. Previous research has shown that motivational and personal resources, such as the facets of PsyCap, can positively influence one’s career success [17,18] and reduce career insecurities [42,43,44]. For instance, a recent study by Kauffeld and Spurk [17] found a positive relationship between PsyCap and career success. Similarly, Darvishmotevali and Ali [42] found a positive moderating effect of PsyCap on job insecurity, reporting that PsyCap increases stress tolerance and positive perception about the events. Thus, we suggest that individuals’ high personal resources (i.e., high PsyCap) can compensate (i.e., mitigate) for the negative effects of career insecurity on career success. Hence, we state the following hypothesis:

**Hypothesis** **3.** *PsyCap moderates the relationship between career insecurity and career satisfaction. This relationship is weaker or not significant when PsyCap is high (3a) and stronger when PsyCap is low (3b).*

### 2.5. The Mediating Role of Career Satisfaction

COR theory [15] posits that resource gain facilitates negative employee experiences by boosting skills and knowledge and reducing stress and strain through further conservation of resources. Accounting for the assumed moderating role of PsyCap on career insecurity [42,43,44], we expect that employee career satisfaction benefits from this relationship. With increased career satisfaction through PsyCap, employees should report fewer worries about their careers during the COVID-19 pandemic. This, in turn positively impacts the worries caused by the COVID-19 pandemic. Research has shown that career satisfaction can indeed reduce career-related outcomes, such as employee turnover intentions [45], or increase perceived organizational support [46]. Therefore, satisfied with their careers, we assume that employees would express fewer worries about their careers due to the COVID-19 pandemic. Hence, we propose the following hypotheses:

**Hypothesis** **4.** *Career satisfaction mediates the effect of career insecurity on career-related COVID-19 worries.*

Taken together, because of the purported role of PsyCap on career-related outcomes, such as career success [17,18] or career insecurities [42,43,44], our major expectation is that PsyCap alleviates the effects of employee career insecurities on their career satisfaction. Therefore, we suggest the following:

**Hypothesis** **5.** *The indirect effect of career insecurity on career-related COVID-19 worries depends upon different levels of PsyCap. This indirect effect is significant at the low levels of PsyCap but not at the high levels of PsyCap.*

**Figure 1:**
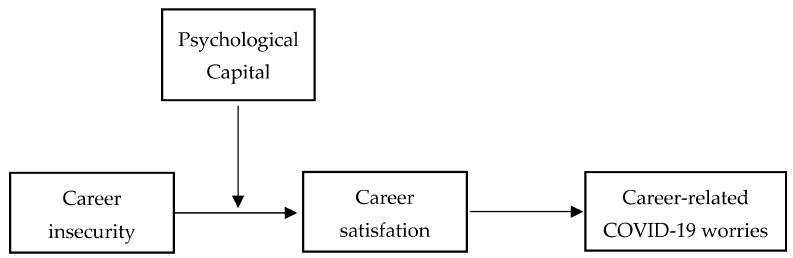
Research model.

To look into the relationships between our study variables, see the hypothesized research model in Figure 1.

## 3. Materials and Methods

### 3.1. Sample

We conducted an online study during the summer and autumn of 2021 and recruited the sample through Sosci Panel (www.soscipanel.de). The panel includes German-speaking participants and utilizes the convenience sampling technique. Voluntarily, 310 and 303 completed the survey at times 1 (T1) and 2 (T2), respectively. However, our final sample included 185 participants who completed the survey at T1 and T2 (81 females). The age ranged from 19 to 67 (M*_age_* = 43.61, SD*_age_* = 12). The majority of the participants held a bachelor’s or equivalent degree (40.54%), lived with a partner (43.24%), had no children (61.08%), worked full-time (67.02%), and worked one day per week from home (37.30%). On average, participants have worked in their companies for 12.84 years (SD = 10.49). Our data and analyses procedure are available in the Open Science Framework (OSF): https://osf.io/3fdr7/ (accessed on 20 November 2022).

### 3.2. Measures

#### 3.2.1. Career Insecurity

We measured career insecurity via a 4-item measure developed by Höge et al. [47]. Participants responded on a 6-point rating scale ranging from 1 (*completely disagree*) to 6 (*completely agree*). Sample item includes “I am not sure whether I shall achieve my career aims”. Cronbach’s alphas were 0.74 (T1) and 0.72 (T2).

#### 3.2.2. Psychological Capital

We used the 12-item short version of the Psychological Capital Questionnaire (PCQ, [12]) to measure psychological capital, which is a robust measure [17]. The scale assesses all four mechanisms of PsyCap: 3 items for efficacy (e.g., “I feel confident in representing my work area in meetings with management”), 4 items for hope (e.g., “If I should find myself in jam at work, I could think of many ways to get out of it”), 3 items for resilience (e.g., “I usually take stressful things at work in stride”), and 2 items for measures optimism (e.g., “I always look on the bright side of things regarding my job”). Participants answer on a 6-point rating scale ranging from 1 (*strongly disagree*) to 6 (*strongly agree*). Because we focused on the unified scale and not dimensions, we averaged the responses on the 12 items to attain PsyCap. Cronbach’s alpha of the unified scale was 0.87 (T1) and 0.88 (T2).

#### 3.2.3. Career Satisfaction

We measured participants’ subjective career success using a German version of the career satisfaction scale [48]. Participants answered on a 5-point rating scale ranging from 1 (*completely disagree*) to 5 (*completely agree*). A sample item is “I am satisfied with the progress I have made toward meeting my overall career goals”. Cronbach’s alphas were 0.90 (T1) and 0.91 (T2).

#### 3.2.4. Career-Related COVID-19 Worries

To measure career-related COVID-19 worries, we adapted one item used by recent research [17], asking participants to answer this question: “All in all, how worried are you about losing your job because of COVID-19?” The response form ranges from 0 (*not worried at all*) to 100 (*worried completely*). Previous research has shown that the single-item measure can often suffice for simple (i.e., one-dimensional) easily understood constructs [49]. For instance, recent research argues that, although single-item measures might suffer from measurement error or reliability, they can be valid reflections of the underlying construct, especially when the psychological construct is not complex [50]. Research has a long history of using single-item measures as valid and reliable instruments, such as the global job satisfaction scale [51,52,53].

### 3.3. Data Analysis

Before testing our hypotheses, we conducted several multi-group confirmatory factor analyses (CFAs) on the items included in our study. We loaded all items on a single factor in the first model and used time (T1 and T2) as the group variable. Next, we modeled our main variables as individual factors and loaded corresponding items on each of them. At this step, we modeled psychological capital as a higher-order factor with its four dimensions and added time (T1 and T2) as the group variable. We compared the two models to decide on the better-fitting model. Additionally, we tested measurement invariance across time by constraining only factor loading to be equal across time (weak invariance) and by constraining intercepts and factor loadings to be equal across time (strong invariance). We compared these steps to decide if adding the constraints significantly worsens the model.

To test our hypotheses, we first conducted correlation analyses to test the first two hypotheses. Afterward, we ran a moderation analysis with career satisfaction measured at T2 as an outcome variable, PsyCap, career insecurity measured at T1, and the interaction between the two as a predictor. We also added the control variables of age, sex, career satisfaction, PsyCap, and career insecurity measured at T1. Furthermore, we tested if career satisfaction mediates the relationship between career insecurity and career-related COVID-19 worries by conducting a mediation analysis. Here, we regressed career satisfaction measured at T2 on career insecurity measured at T1 while controlling for career satisfaction measured at T1. Similarly, we regressed career-related COVID-19 worries measured at T2 on career satisfaction measured at T1 while controlling for career insecurity measured at T1. Finally, we investigated if the mediation effect depends on the different levels of PsyCap by adding it at T1 as a moderator in the mediation analysis.

## 4. Results

### 4.1. Confirmatory Factor Analyses

We compared different measurement models for the latent constructs aligning with the assumed model (i.e., best fit). The results of CFA showed that compared with the one-factor model, the four-factor model with PsyCap as a higher-order factor showed a better model fit (χ² = 762.43, df = 400, CFI = 0.91, TLI = 0.89, RMSEA = 0.07, 95% CI = 0.062 to 0.077, SRMR = 0.069), which is the theoretically implied model. Furthermore, we investigated if constraining some parameters of the three-factor model to be equal across the group (across time) significantly improved or worsened our model fit. As shown in Table 1, constraining factor loadings (i.e., weak invariance) or constraining factor loadings and intercepts (i.e., strong invariance) did not significantly change the model fit. Thus, we concluded the models to be equivalent across time points (see Table 1).

### 4.2. Descriptive Statistics and Correlations

The descriptive statistics and correlations among study variables are shown in Table 2. As shown, career insecurity and PsyCap measured at T1 are significantly correlated with career satisfaction measured at T2 (*r* = −0.47, *p* < 0.01 and *r* = 0.40, *p* < 0.01, respectively). Furthermore, career insecurity, PsyCap, and career satisfaction measured at T1 are significantly associated with career-related COVID-19 worries measured at T2 (*r* = 0.27, *p* < 0.01, *r* = −0.21, *p* < 0.01 and *r* = −0.16, *p* < 0.05 respectively). These results support our Hypotheses 1 and 2.

### 4.3. Moderating Effect of Psychological Capital

**Figure 2:**
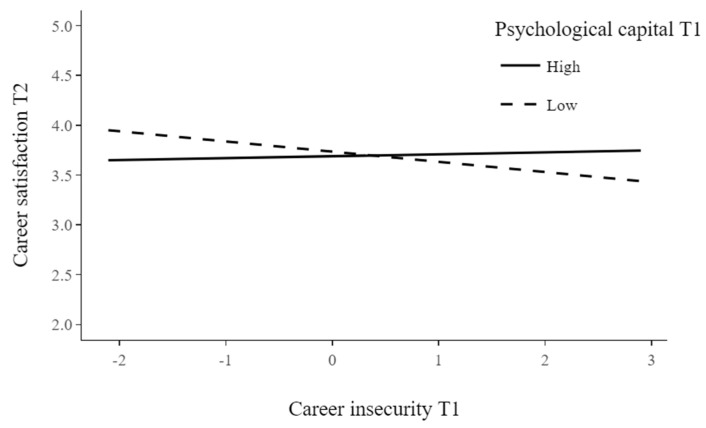
Regression slopes for the interaction of psychological capital and career insecurity on career satisfaction.

To investigate if PsyCap significantly moderates the effect of career insecurity on career satisfaction, we ran a moderation analysis. The analyses yielded a significant interaction effect of PsyCap and career insecurity measured at T1 on career satisfaction measured at T2 (*b* = 0.08, *t* = 2.21, *p* < 0.05). Simple slope analyses showed that for high levels of PsyCap (i.e., mean + standard deviation), the relationship between career insecurity measured at T1 and career satisfaction measured at T2 was not significant (*b* = 0.02, *t* = 0.40, *p* > 0.05). However, for low levels of psychological capital (i.e., mean—standard deviation), the relationship was significant (*b* = −0. 10, *t* = −2.27, *p* < 0.05). These results support Hypothesis 3 (see Figure 2).

### 4.4. Career Satisfaction as a Mediator

**Figure 3:**
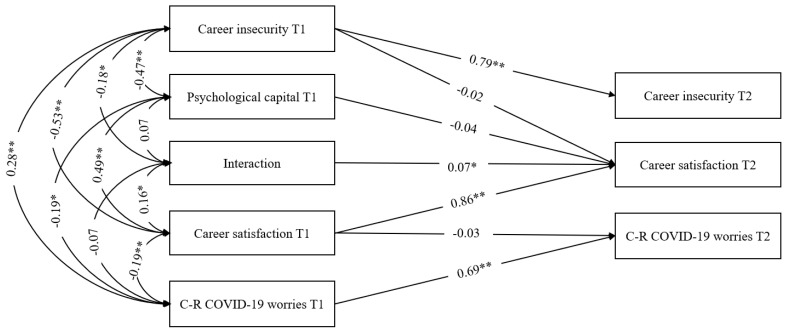
Regression path analysis on effects between study variables in both times (T1–T2) and the interaction effect. Interaction denotes the interaction between psychological capital and career insecurity measured at T1. C-R: Career-related. * *p* < 0.05. ** *p* < 0.01.

We further investigated if career satisfaction mediates the relationship between career insecurity and career-related COVID-19 job worries. The results of the mediation analysis showed that the indirect effect of career insecurity on career-related COVID-19 worries through career insecurity was not significant (*b* = 0.01, *p* > 0.05, 95% CI [−0.13, 0.19]). As a final step, we investigated if this indirect effect depends upon different levels of PsyCap. Hence, we ran a moderated mediation analysis. The results showed that the indirect effect of job insecurity on career-related COVID-19 worries though career satisfaction depended upon neither low (i.e., mean − standard deviation) nor for high-levels (i.e., mean + standard deviation) of PsyCap (*b* = 0.07, *p* > 0.05, 95% CI [−0.21, 0.45], and *b* = −0.04, *p* > 0.05, 95% CI [−0.35, 0.19], respectively). With these results, we could not find support for Hypotheses 4 and 5 (see Figure 3).

## 5. Discussion

The present study sought to investigate the role of career insecurity in employees’ career satisfaction (i.e., subjective career success) and the subsequent career-related COVID-19 worries. Results revealed a positive association between career insecurity and career satisfaction and career-related COVID-19 worries, supporting both Hypotheses 1 and 2. In addition, our major aim was to look into the moderating role of PsyCap between career insecurity and career satisfaction. Moderation analysis supported our hypotheses (3a-3b), revealing that a high PsyCap can significantly mitigate the possible negative effects of career insecurity on employees’ career satisfaction. Finally, we hypothesized that career satisfaction would mediate the negative effects of career insecurity on employees’ career-related COVID-19 worries and that the indirect effect of career insecurity on career-related COVID-19 would depend upon different levels of PsyCap. However, the results did not support these expectations and rejected Hypotheses 4 and 5.

Research on career success has long noted the positive implications of a satisfying career [26,54] and the effects of possible negative life events that can threaten it [2,4]. However, with the current ongoing developments, due to flexibility and technological advancements [55,56], as well as crisis, such as the COVID-19 pandemic [57], career changes are inevitable, becoming less secure, which can be perceived as a threat to employees’ career success [10]. In line with these findings, our study showed that career insecurity can be a threat to employee career satisfaction. Moreover, our findings are especially applicable to crises such as the COVID-19 pandemic, where employees experience massive changes in their careers, with some switching between home and office [58,59] and others going jobless [60].

Related to factors that can mitigate the negative effects of career insecurity on employees’ career satisfaction, we employed PsyCap as a personal resource. Findings show that PsyCap can play an essential role in employees’ subjective and objective career success [17,61] and many other social- and health-related outcomes [62,63], even in the context of COVID-19. Furthermore, PsyCap represents a positive psychological state, facilitating employees’ experiences during challenging and uncertain times [64,65]. For example, PsyCap has been shown to have positive effects on employees’ employability [62,64], reduce stress and turnover [66,67], as well as increase job satisfaction [12]. Our results are in line with these empirical findings, supporting the positive role of PsyCap for employees’ career insecurity. Furthermore, according to COR theory [15], PsyCap mechanisms can play a pivotal role for employees due to their combined effects by acting as a caravan of resources. Similarly, PsyCap authors [13] reported that PsyCap makes up an exceptional resource because of the four critical mechanisms it entails. Hence, employees and organizations at large should utilize our findings and invest in increasing PsyCap facets, which would in turn facilitate their experiences during crises.

In addition to the moderating role of PsyCap, we were interested in looking into the possible effects of career satisfaction (i.e., improved through PsyCap’s role on career insecurity) for employee career-related COVID-19 worries. We assumed that career satisfaction would reduce possible worries caused by the COVID-19 pandemic. However, the results did not confirm our assumptions since career satisfaction did not predict career-related COVID-19 worries. This can imply two important implications: First, results might indicate that even satisfied employees can struggle and feel worried during times of crisis [67]. Whether this impacts their work performance and job-related outcomes are beyond current research power to answer. Work performance can be a good indicator of possible negative ramifications of the worries caused by the COVID-19 pandemic. Second, accounting for the role of PsyCap for career insecurity, our findings suggest that, although worries can be present during times of crises, employees might be satisfied with their career because of, for example, their future temporal focus [16], which can be explained by the mechanisms of PsyCap such as hope and optimism. In fact, PsyCap correlated negatively with career-related worries, which suggests a positive role in this outcome. Similarly, findings show that resilience can increase employee satisfaction and well-being, even during crises and turbulences [67,68,69].

### 5.1. Practical Implications

A Chinese proverb says, “even the cleverest housewife cannot cook without rice”. Our main aim in this research was to show the positive implications that a high PsyCap can yield for employees’ experiences (i.e., career satisfaction), especially in times of turbulence and crises, such as the COVID-19 pandemic. Our study showed that PsyCap can be a critical personal resource for employees during challenging times. Hence, as the Chinese proverb goes, employees and organizations must pay attention to PsyCap and work on its development. Accounting for the four mechanisms of PsyCap (e.g., self-efficacy), we adhere to the previous recommendations [11] for organizations to utilize approaches provided by the authors of self-efficacy on the improvements of one’s self-belief [70] as a critical facet of PsyCap. According to Bandura, employees can increase their efficacy by mastering experiences, learning from role models, or through others’ support and persuasion. Similarly, to increase reliance, organizations can employ training programs that can enhance employee compassion, such as compassion cultivation training (CCT, [71]).

In addition, our findings suggest another important implication about the role of career satisfaction for employees’ worries in uncertain and challenging environments. In line with our results, employees and organizations should consider that one’s satisfaction does not denote that employees feel less worried. This is especially important in the context of our study. Hence, organizations should support employees and provide ways to mitigate their worries that uncertain environments, such as the COVID-19 pandemic, might yield. Recent findings show that traits such as emotional stability can mitigate the negative effects of COVID-19 worries [11]. Organizations can use ways to develop and strengthen emotional stability, such as the emotion regulation strategies developed by Gross [72,73]. For instance, reappraisal (an emotion regulation strategy) reduces anxiety and worries [74,75]. A recent study showed that emotion regulation strategies alleviate symptoms and difficulties, such as anxiety [76]. Thus, by enhancing one’s emotion regulation skills, employee career satisfaction might also benefit by reduced worries and anxiety, especially in a context such as the COVID-19 pandemic, where stress and worries are inevitable. These suggestions align with the COR theory, which suggests that boosting resources can be a robust approach to strengthening employee skills [15].

### 5.2. Limitations and Future Research

Concerning limitations and future research, we acknowledge a couple of important points. First, our sample was German-speaking, and any generalization should account for this. Hence, future research must replicate our findings with a different and possibly more varied sample. Second, because we were interested to learn about the role of career insecurity and PsyCap during the COVID-19 pandemic, this might limit generalizations in other contexts. Therefore, it might be crucial to examine whether this role of PsyCap can be replicated in various contexts. Nevertheless, with careers changing vastly due to globalization, flexibility, and similar factors, we presume that this role of PsyCap should remain relatively static because PsyCap is shown to be a robust and stable resource. Moreover, despite the robust design of our study, one should be cautious when drawing causal claims, especially about the intraindividual effects. Therefore, it might be helpful if future research investigates current findings, tests all measures in different data collection points, and looks into career trajectories related to PsyCap, career insecurity, and career satisfaction. Finally, future research should strive to replicate our study with a larger sample. We conducted a sensitivity power analysis to examine the effect size that is detectable by our sample size. With the correlations between our study variables and our sample size, we found that our sample size can detect an indirect effect of 0.08, which is smaller than what we found. This might be why the indirect and moderated indirect effects were not significant in our study. Hence, future studies can replicate our findings with a more powered sample.

## 6. Conclusions

Our findings revealed that career insecurity can negatively affect employees’ career satisfaction. However, as results suggest, PsyCap can mitigate this negative effect of career insecurity on career satisfaction. PsyCap is a robust personal resource in career research, and results show that it can be a promising intervention for employee careers, especially in challenging environments. Therefore, organizations must increase employee PsyCap, which would facilitate their experiences during difficult times.

## Figures and Tables

**Table 1:** Fit indices with different factor solutions for the study variable and measurement invariances.

Models	*χ²*	*df*	Δ*df*	Δ*χ²*	CFI	TLI	RMSEA	SRMR	AIC	BIC
One factor model	1819.44	418	-	-	0.64	0.61	0.14	0.11	24,861.30	25,377.88
Four factors model	762.43	400	18	1057 ***	0.91	0.89	0.07	0.069	23,840.28	24,427.31
Measurement invariance										
Configural invariance	762.43	400	-	-	0.91	0.89	0.07	0.069	23,840.28	24,427.31
Weak invariance	785.87	418	18	23.44	0.91	0.90	0.069	0.074	23,827.72	24,344.30
Strong invariance	803.15	432	14	17.28	0.91	0.90	0.068	0.075	23,817.01	24,278.80

*N* = 185. Factors: career insecurity, psychological capital, subjective career success, and job-related COVID-19 worries (only one item). *χ²*: chi-square; *df*: degrees of freedom; CFI: comparative fit index; TLI: Tucker–Lewis index; RMSEA: root mean square error of approximation; SRMR: standardized root mean square residual; AIC: Akaike information criterion; BIC: Bayesian information criterion. *** *p* < 0.001.

**Table 2:** Means, standard deviations, and correlations among study variables.

Variable	*M*	*SD*	1	2	3	4	5	6	7	8	9
1. Sex (1 = female, 2 = male)	-	-									
2. Age	43.58	12.02	−0.23 **								
3. Career Insecurity T1	3.10	1.09	0.07	−0.34 **							
4. Career Insecurity_T2	3.10	1.05	0.10	−0.27 **	0.79 **						
5. Psychological Capital T1	4.63	0.77	−0.06	0.13	−0.47 **	−0.43 **					
6. Psychological Capital T2	4.66	0.74	−0.07	0.13	−0.52 **	−0.58 **	0.77 **				
7. Career Satisfaction T1	3.70	0.79	−0.07	0.03	−0.53 **	−0.50 **	0.49 **	0.51 **			
8. Career Satisfaction T2	3.68	0.84	−0.02	−0.03	−0.47 **	−0.49 **	0.40 **	0.53 **	0.86 **		
9. C-R COVID-19 Worries T1	9.56	21.15	0.04	−0.08	0.28 **	0.30 **	−0.19 *	−0.18 *	−0.18 *	−0.20 **	
10. C-R COVID-19 Worries T2	9.81	21.41	−0.06	−0.01	0.27 **	0.30 **	−0.21 **	−0.18 *	−0.16 *	−0.23 **	0.70 **

T1: Time 1; T2: Time 2. C-R: Career-related. * *p* < 0.05. ** *p* < 0.01.

## Data Availability

The data materials are available in the OSF: https://osf.io/3fdr7/ (accessed 20 November 2022).
